# Evidence of bidirectional transmembrane signaling by the sensor histidine kinase GacS from *Pseudomonas aeruginosa*

**DOI:** 10.1016/j.jbc.2025.108521

**Published:** 2025-04-23

**Authors:** Safoura Salar, Steve Silletti, Florian D. Schubot

**Affiliations:** 1 Department of Biological Sciences, Virginia Polytechnic Institute and State University, Blacksburg, Virginia, USA; 2 Department of Chemistry and Biochemistry, University of California, San Diego, California, USA

**Keywords:** bacterial signal transduction, histidine kinase, biofilm, protein-protein interaction, allosteric regulation, phosphorelay, two-component signaling, multi kinase networks

## Abstract

Membrane-embedded signaling histidine kinases (SKs) from two-component and phosphorelay signal transduction systems play central roles in the gene regulation of bacteria, fungi, and plants. The SK GacS is a global regulator of gene expression in the human pathogen *Pseudomonas aeruginosa*. The interactions between GacS and another SK, RetS, are a model for studying non-canonical crosstalk in multikinase networks. During planktonic growth, RetS inhibits GacS to upregulate expression of virulence factors associated with acute *P*. *aeruginosa* infections and repress genes linked to chronic infection. Conversely, GacS activation promotes biofilm formation and chronic infection but suppresses factors required during acute infection. Using a combination of hydrogen-deuterium exchange mass spectrometry (HDX-MS) and mutational analysis in conjunction with functional assays, we show that binding of an extracellular ligand promotes GacS signaling through two mechanisms: (1) by increasing GacS autokinase activity and (2) by decreasing the affinity between GacS and RetS. Intriguingly, RetS binding to the intracellular histidine kinase domain of GacS also triggered conformational changes in the extracellular sensory domain of GacS. This allosteric effect was confirmed in a biochemical assay, showing RetS increases the affinity of a chimeric CitAGacS receptor for citrate by almost tenfold. This finding establishes the first precedent of inside-out cross-membrane signaling in SK systems. Taken together, our data are consistent with a model wherein RetS binding primes GacS for signal sensing during planktonic growth. Binding of the unknown ligand at the onset of biofilm formation causes dissociation of the RetS-GacS complex to lock GacS in a kinase ON conformation.

Two-component and phosphorelay signal transduction systems are the primary means whereby bacteria adapt gene expression patterns to complex environmental conditions. In a classical two-component system, ligand binding to the extracytoplasmic sensory domain of the sensor histidine kinase (SK) facilitates autophosphorylation of a conserved histidine residue located within dimerization and histidine phosphotransfer (DHp) domain by the catalytic (CA) domain using ATP as a substrate. Canonical SKs are dimeric and autophosphorylation usually occurs *in trans* (1). The initial autophosphorylation step is followed by transphosphorylation of a conserved aspartic acid residue in a cognate response regulator (RR) (2, 3). Canonical RRs are transcription factors that dimerize upon phosphorylation to enhance their DNA binding affinity. The closely related phosphorelay systems employ two additional phosphotransfer steps mediated by distinct receiver (REC) and Histidine Phosphotransfer (HPt) proteins. In unorthodox SKs, the latter two functions are integrated into the SK domain architecture, whereas hybrid SKs contain one or more REC domains, but no HPt domain (4).

Because evolutionary analyses had suggested that crosstalk between SKs is generally not favored, SKs were once thought to operate exclusively along independent linear phosphate-transfer chains. Over the past 15 years, the original paradigm was revised following the discovery of webbed multikinase networks (MKNs) that coordinate the expression of hundreds of genes (5, 6). The stringent selectivity requirements were found to be more relaxed among unorthodox and hybrid SKs (7). Frequently, underlying such crosstalk is a simple broadening of the substrate specificity, such as the phosphorylation of an RR by a non-cognate SK or HPt domain (5, 6). However, other interactions involve novel mechanisms that have yet to be fully elucidated. In particular, observed direct interactions between the histidine kinase regions of two distinct SKs are difficult to reconcile with our current understanding of dimeric SK structures. The first MKN to be discovered encompassed the three *Pseudomonas aeruginosa* SKs, GacS, LadS, and RetS (8, 9, 10). This network has now been expanded to at least seven SKs. Together, these enzymes orchestrate the transition between motile and sessile *P*. *aeruginosa* lifestyles (8, 9, 11, 12). These distinct lifestyles not only involve vastly different gene expression patterns but are also linked to distinctive human infections. Acute *P*. *aeruginosa* infections, occurring in immunocompromised individuals such as transplant patients, are associated with an upregulation of genes required for motility, such as swimming, swarming, and twitching, and the expression of the type III secretion system (T3SS). In contrast, *P*. *aeruginosa* biofilms, characteristic of chronic infections, entail elevated production of the exopolysaccharides *Psl*, alginate, and *Pel*, as well as the activation of the type VI secretion system (T6SS) (8, 13, 14, 15, 16). Chronic *P*. *aeruginosa* infections are a major complication in cystic fibrosis patients (13, 14, 17, 18).

The widely conserved GacS/GacA phosphorelay system controls the Gac/Rsm (Global activation of Antibiotic and Cyanide synthesis) pathway. Across bacterial taxa, this pathway has undergone a remarkable functional diversification, adapting to assume various roles in regulating cellular processes (19, 20, 21). In *P*. *aeruginosa*, the Gac/Rsm pathway controls the expression of more than 500 genes to promote biofilm formation while suppressing the expression of gene clusters linked to motility and acute infection (22). During planktonic growth, GacS signaling is strongly inhibited by the SK RetS (5, 8, 23). *retS*^*-*^ *P*. *aeruginosa* strains are avirulent and display a hyperbiofilm phenotype (10). RetS employs three distinct mechanisms to inhibit GacS signaling (8, 23). The initial GacS autophosphorylation step is blocked through direct binding interactions between the histidine kinase (HK) regions of RetS and GacS, while the other two mechanisms target downstream phosphotransfer events (8, 23). We have shown that RetS blocks GacS *trans*-autophosphorylation by replacing the canonical homodimeric DHp-DHp subdomain interface of GacS with a novel heterodimeric RetS-GacS DHp-DHp interface (24, 25). At the onset of biofilm formation, GacS is hypothesized to be activated through binding of an unknown extracellular signal to its periplasmic PAS-like PDC domain (GacS_PDC_) (26, 27). GacS_PDC_ is required for GacS activation, but the precise role of the unknown ligand remains to be established (28). The extracellular sensory domain of RetS binds at least two distinct ligands with opposite effects. A ligand originating from *P*. *aeruginosa* itself causes activation of the Gac/Rsm pathway (29) while a ligand associated with the mucins found in human lung tissues, inhibits the same pathway (30). The identities of the RetS ligands are still not known but they are likely carbohydrate-based moieties (30, 31).

Initially, RetS binding was proposed to cause the complete dissociation of the GacS homodimer (8). Our structural and biochemical studies indicate that GacS retains its dimeric structure through interactions in the membrane-proximal GacS HAMP domain (GacS_HAMP_) even in the absence of its transmembrane and periplasmic domains (25). However, in a recent report, RetS_HK_ binding was observed to cause a full dissociation of dimeric GacS-cytosolic region (27). The same study also reported enhanced GacS-RetS affinity in the presence of GacS_HAMP_ and the Signaling helix (S-helix) spanning GacS_HAMP_ and DHp domains (GacS_DHp_). These findings led the authors to propose the RetS-GacS interface extends beyond the DHp-DHp interface initially revealed by other mutational analyses and the crystal structure of the complex between the GacS_DHp_ domain and RetS_HK_ (Fig. 1*A*) (9, 24, 25).

**Figure 1:**
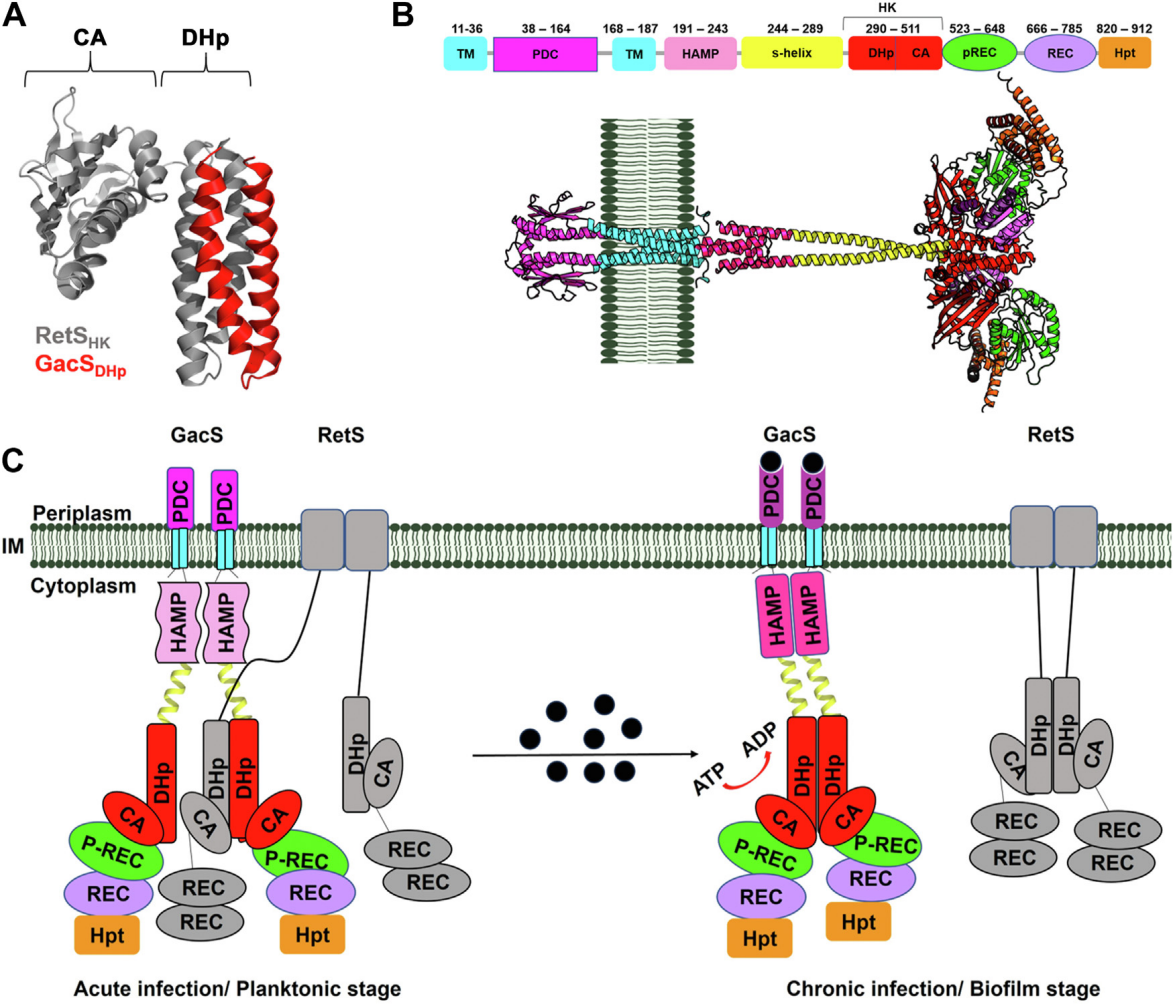
**RetS-GacS interactions**. *A*, crystal structure of a RetS_HK_-GacS_DHp_ complex (pdb code: 7N0E), revealing the heterodimeric DHp-DHp interface. *B*, alphafold2 generated structural models of the GacS dimer. *C*, working model for the regulation of RetS-GacS interactions. Details are provided in the text.

In the present study, we investigate the allosteric consequences associated with the formation of the heteromeric DHp-DHp interface between RetS and GacS. Because modelling suggests a tight conformational coupling between GacS extracellular, transmembrane, HAMP, and histidine kinase regions (Fig. 1*B*), we sought to investigate the possibility that RetS allosterically modulates signal perception in the extracellular GacS_PDC_ domain (Fig. 1*C*). Inside-out signaling has never been reported for an SK system. However, in the distantly related methyl-accepting-chemotaxis proteins (MCPs), receptor affinity for the extracellular ligand is modulated through the intracellular methylation of the MCPs (32, 33, 34, 35).

Through the combination of molecular modeling, hydrogen-deuterium-exchange mass spectrometry (HDX-MS), biochemistry, and microbiology approaches, this study provides novel insights into the interplay between intracellular and extracellular interactions of GacS. In the presence of RetS_HK_, the HDX-MS data show no significant changes at the predicted dimerization interface of GacS in the HAMP and PDC regions that would be consistent with a dissociation of the GacS dimer. Excitingly, however, the HXD-MS data do show an impact of RetS_HK_ binding on the conformations of the GacS transmembrane (GacS_TM_) regions and GacS_PDC_. Because the GacS ligand is unknown, a fully functional CitAGacS chimeric protein was used to facilitate further analyses. In support of a bidirectional signaling model, RetS_HK_ binding was found to enhance the affinity of CitAGacS for citrate almost tenfold. Conversely, citrate binding to CitAGacS was found to weaken RetS-GacS interactions significantly and enhance CitAGacS autokinase activity *in vitro*. Collectively, our results are reconciled in a model that posits three major conformations for GacS with distinctive affinities for its periplasmic ligand and RetS. Because RetS-GacS interactions induce structural changes not only in the native GacS_PDC_ domain but also in the sensory domain of CitA, we speculate that the observed tunability of ligand affinity may be a broadly conserved but understudied phenomenon among SKs.

## Results

### Intracellular RetS-GacS DHp-DHp domain interactions alter the conformation of the distant extracytoplasmic GacS_PDC_ domain

Prior structural and biochemical analyses have mapped the binding interactions between the histidine kinase regions of RetS and GacS to their respective DHp domains and demonstrated that RetS_HK_ is sufficient for GacS binding and for inhibiting GacS autophosphorylation (9, 10, 24, 25). In the crystal structure of the RetS_HK_/GacS_DHP_ complex, the homomeric GacS DHp-DHp interface has been replaced by an analogous heterodimeric GacS-RetS interface (24). This heterodimeric DHp-DHp interface is almost identical to that found in the crystal structure of the homodimeric GacS histidine kinase (24, 27). The RMSD for the superposition of the two four-helix bundles (PDB codes: 7Z8N and 7N0E) is only 0.45 Å (Fig. S1). Because the crystal structure only encompassed the interacting regions, the global structural consequences of RetS-GacS complex formation on the two proteins have not been characterized. To address this gap and study conformational changes associated with the interaction, we expressed and purified a GacS_649_ construct, consisting of all domains required for transmembrane signaling, the histidine kinase region, and pseudo-receiver (p-REC) domain, but lacking the HPt and receiver (REC) domains (Fig. 1*B*). This construct was chosen to focus the analysis on the interplay between RetS_HK_ binding and GacS cross-membrane signaling. HDX-MS analysis was used to measure changes in surface accessibility of the GacS_649_ protein in the presence of RetS_HK_ by detecting altered rates and levels of hydrogen-deuterium (H/D) exchange.

**Figure 2:**
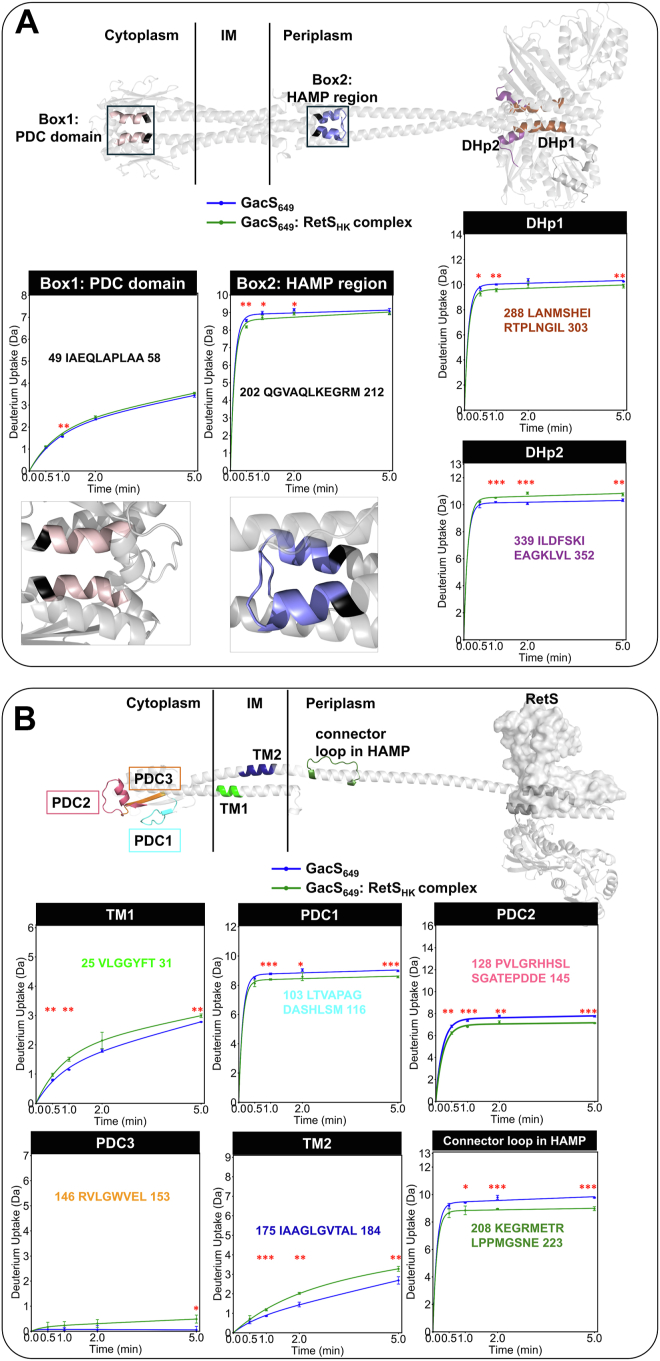
**HDX-MS analysis of effect of RetS**_**HK**_***binding*****on GacS**_**649**_**.***A*, HDX analysis of the impact of RetS_HK_ on GacS_649_ dimerization. The uptake differences are mapped onto the GacS_649_ dimer. Discussed regions and peptides are highlighted. *B*, structural changes in GacS_649_ upon RetS_HK_ binding visualized by HDX-MS analysis. Uptake differences are mapped onto the GacS_649_ monomer. RetS_HK_ was modelled into the binding site using the crystal structure of the RetS_HK_-GacS_DHp_ complex as guide and is displayed as grey solvent accessible surface. ∗*p* < 0.05, ∗∗*p* < 0.01, ∗∗∗*p* < 0.001.

To examine potential structural changes along to GacS dimerization interface in the HDX-MS data, we constructed a model of a GacS_649_ dimer using AlphaFold2 (Fig. S2). Confidence scores of the model were very high with pLDDT scores above 90 for most regions (Fig. S2). The modeled interface closely matched the dimerization interface of the GacS_HK_ crystal structure (6) (the RMSD for the superimposed C-alpha atoms encompassing residues 236–512 of the histidine kinase and S-helix regions was 2.7 Å). Similarly, the predicted dimerization interfaces of the HAMP domain and the GacS periplasmic domain closely mirrored those of known crystal structures in homologous domains (36, 37, 38). We first examined the DHp-DHp interface, which was not necessarily expected to show significant change, because the homomeric and heteromeric dimerization interfaces are so similar. In the crystal structures, differences between the two complexes are only apparent at the carboxy-terminal end of the DHp domain of GacS, around residues 339 to 350 (Fig. S1). This difference is also captured in the HDX-MS data, where the peptides of amino acids 288 to 303 and 339 to 352, respectively, representing the interacting N-terminal and C-terminal residues of GacS_DHp_, show small but statistically significant differences in deuterium uptake in the GacS_649_-RetS_HK_ complex (Fig. 2*A*). Next, we investigated the impact of RetS_HK_ on peptides representing the putative GacS dimerization interface in the GacS HAMP and PDC regions. None of the representative peptides displayed increased solvent accessibility in the presence of RetS_HK_. For example, residues 49 to 58 and 128 to 145 belong to two α-helices predicted to participate in the dimerization of the PDC domain. A peptide encompassing residues 49 to 58 shows a low maximum uptake level of 42% and H/D exchange levels remain unchanged in the presence of RetS_HK_ (Fig. 2*A*). The peptide containing amino acids 128 to 145 shows a maximum uptake of just about 43% and RetS_HK_ actually causes a small decrease in solvent accessibility, consistent with a slight compaction of the region (Fig. 2*B*). Peptides 185 to 199 (Fig. S3) and 202 to 212 (Fig. 2*A*) representing the helical regions of GacS_HAMP_, also show no significant changes at the predicted dimerization interface. The GacS S-helices, spanning the GacS_HAMP_ and the DHp domains, also displayed no significant changes in backbone solvent accessibility. H/D-exchange is consistently higher along the entire S-helix, suggesting that intermolecular interactions are weak or absent in this region even prior to RetS_HK_ binding. Collectively, the HDX-MS data therefore do not support a model wherein the GacS dimer is fully dissociated by RetS binding.

Although the dimerization interface of GacS_649_ was largely unaffected, the HDX-MS data did reveal numerous conformational changes in other regions: RetS_HK_ binding significantly impacted GacS residues 351 to 361, which belong to a loop connecting DHp and CA subdomains (Fig. S3). RetS_HK_ also caused a lowered exchange in peptides composed of residues 208 to 223 and 208 to 224, respectively, which represents the connector loop between the two HAMP domain helices (Figs. 2*B*, and Fig. S3). RetS_HK_ binding not only impacts GacS_HAMP_ but also the solvent accessibility for GacS peptides V25-T31 and I175-L184, respectively representing TM helices 1 and 2 (Fig. 2*B*). This increase in the H/D exchange of these peptides contrasts with the decreases that were observed later on in the same regions when citrate bound to CitAGacS_649_ (see below). In addition, small but significant H/D exchange differences map to the putative ligand binding site within the extracellular GacS_PDC_. Although PAS/PDC domains display diverse ligand specificities, the binding pocket is conserved along the face of a β-sheet (44, 45, 46, 47). RetS_HK_ causes a measurable reduction in the exchange indicative of a more compact conformation in peptide L103-M116, which is predicted to form a loop region that folds over a bound ligand (Fig. 2*B*). A peptide containing residues R146-L153 represents the central beta strand at the heart of the putative ligand binding pocket. This peptide shows an increase in solvent accessibility (Fig. 2*B*). Additional GacS_PDC_ peptides also affected by RetS_HK_ binding are presented in Figure S2. Collectively, the observed structural changes in GacS_HAMP_, and TM regions, as well as in GacS_PDC_, support the hypothesis that, despite being separated from the sensory domain by more than 100 Å, the formation of the RetS-GacS interface allosterically regulates the binding of the GacS_PDC_ ligand.

### Ligand binding to the PDC domain decreases the affinity between CitAGacS_649_ and RetS_HK_ and moderately increases GacS autokinase activity

**Figure 3:**
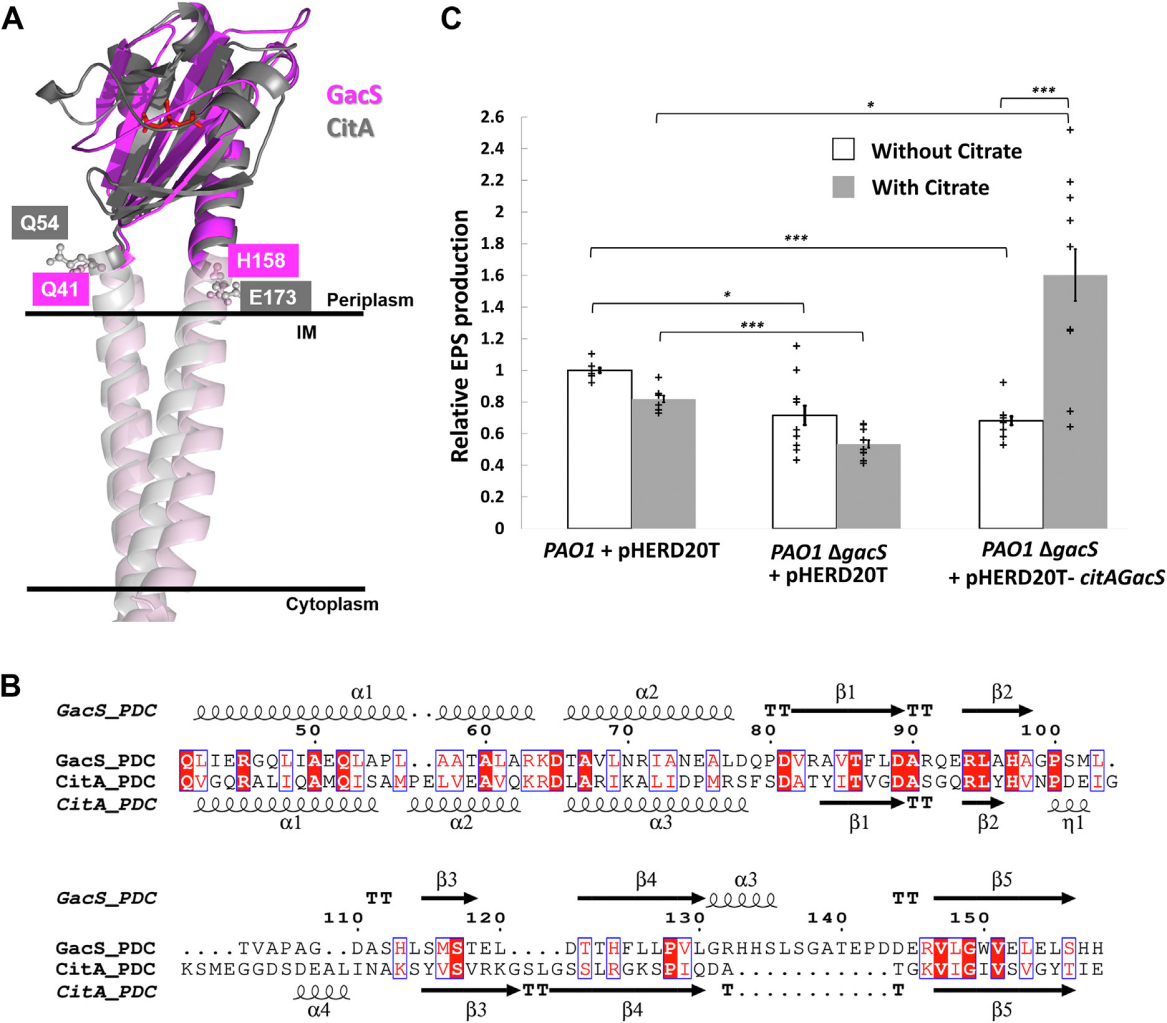
**Design and validation of the CitAGacS chimeric protein.***A*, superposition of the AlphaFold2 models of GacS_PDC_ (magenta) and the CitA_PDC_ (grey). The experimental structure for the CitA domain in complex with citrate is identical to the model (pdb-code 1P0Z). The citrate molecule is indicated as red ball-and-stick-molecule to mark the binding pocket. Selected junction residues are also shown as ball-and-stick. *B*, structure-based alignment of CitA and GacS PDC domains based on the CitA_PDC_ crystal structure and the AlphaFold2-generated model for GacS_PDC_. *C*, Adherence assay monitoring *P*. *aeruginosa* attachment. Wild-type and ΔgacS *P*. *aeruginosa* PAO1 strains contain the empty pHERD20 T plasmid. citAgacS was cloned into the same plasmid behind an arabinose inducible promoter. ∗*p* < 0.05, ∗∗*p* < 0.01, ∗∗∗*p* < 0.001.

The GacS_PDC_ domain is required for GacS activity *in vivo* (28). However, its specific mechanism of action has not been established because the GacS ligand has not been identified. It is possible that, like in canonical SKs, the GacS ligand stimulates GacS autokinase activity. Alternatively, or in addition, the ligand may alleviate GacS inhibition by directly disrupting the interaction between RetS and GacS. To address this question, a chimeric CitAGacS protein was constructed, wherein the GacS_PDC_ domain was swapped against the PDC domain of the citrate sensor CitA from *Klebsiella pneumoniae*. Junction residues were identified through molecular modeling: PDC domains fall under the umbrella of the prevalent PAS domain family (44, 45, 48, 49). CitA_PDC_ possesses a typical PAS fold (47). The NMR structure of GacS_PDC_ displays an “atypical PDC/PAS-like domain fold” (28) that aligns poorly with the CitA_PDC_ structure. Several unusual features of the GacS_PDC_ NMR structure might be indicative of misfolding, perhaps, because it requires the remainder of the protein for stability. These unusual features include large unstructured sections in the middle of the domain, absence of several strands from the usually conserved beta sheet, and a partially buried putative dimerization site in the model. Due to these concerns, an AlphaFold2^38^ model of the dimeric GacS_PDC_ domain in the context of full-length GacS was used for the modeling work rather than the NMR structure. In the predicted model, the GacS_PDC_ dimer closely resembles the CitA_PDC_ structure with an RMSD of 2.3 Å for the aligned 120 residues (Fig. 3*A*). Local structural superposition aligned Q41 of GacS and Q54 of CitA at the amino-terminal junction of the PDC domains, while H158 of GacS and E173 from CitA were selected as the carboxyterminal junction (Fig. 3, *A* and *B*). All other GacS domains, including both TM helices, were retained in the chimeric protein to replicate signaling of native GacS as closely as possible. Full-length CitAGacS was expressed *in trans* in a *gacS*^-^ PAO1 strain to determine if the protein may be activated by citrate *in vivo*. The addition of citrate induced a 1.8-fold increase in EPS production in the complemented strain compared to the wild-type PAO1 strain (Fig. 3*C*). Because RetS acts exclusively through GacS, a *retS*^-^ PAO1 strain is considered representative of “activated GacS” (50). Under the same assay conditions, the *retS*^-^ PAO1 strain also shows 1.8-fold higher levels of EPS than wild-type PAO1 (Fig. S4), suggesting CitAGacS activation levels are representative of those of native GacS.

**Figure 4:**
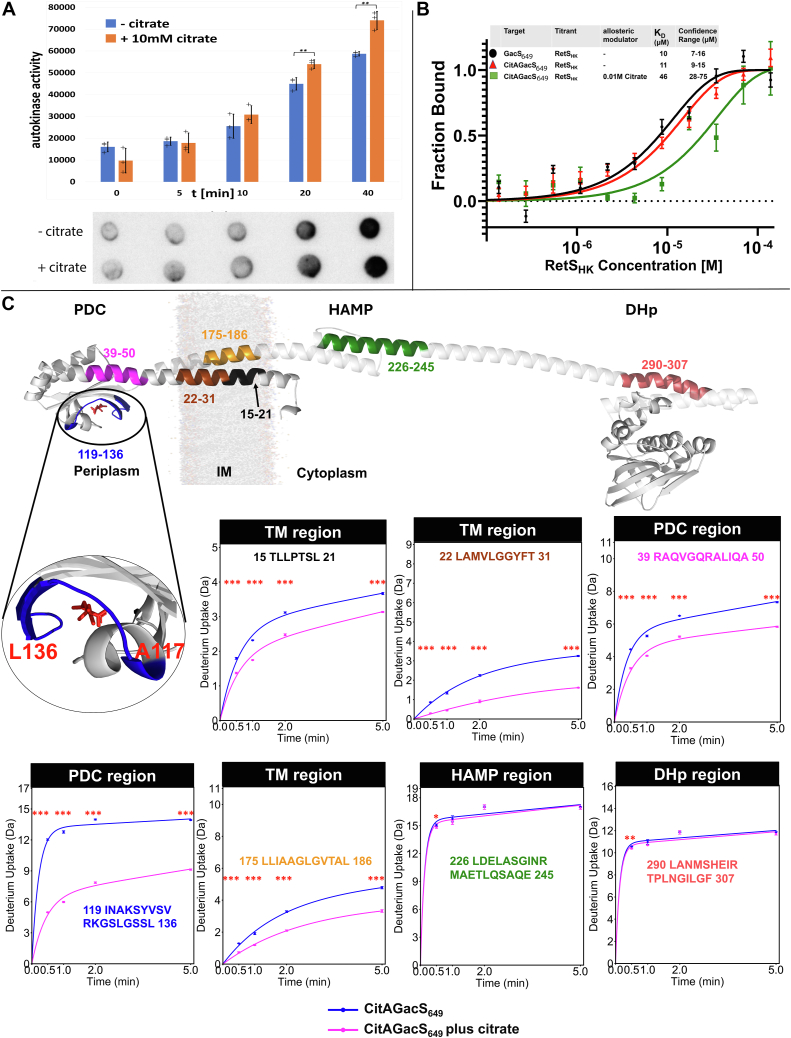
**Analysis of interactions between citrate and CitAGacS**. *A*, Autophosphorylation assay results of the impact of citrate on CitAGacS autokinase activity. The experiments were carried out in triplicate. Error bars indicate the standard deviation (SD) of each measurement. ∗*p* < 0.05, ∗∗*p* < 0.01, ∗∗∗*p* < 0.001. *B*, MST binding experiment of RetS_HK_ to either GacS_649_ or CitAGacS_649_ in the presence or absence of citrate. Each data point represents the average of triplicate measurements. Error bars indicate the standard deviation (SD). *C*, HDX analysis of the interactions between CitAGacS_649_ and citrate. Discussed peptides are highlighted in the model of a CitAGacS_649_ monomer.

The impact of citrate on the autokinase activity of CitAGacS was measured *via* an *in vitro* dot blot autoradiography assay. Addition of 10 mM citrate caused a slight but statistically significant increase in autokinase activity after 20 min (Fig. 4*A*). However, the enzyme had significant activity in the absence of citrate as well, indicating that GacS ligand may not be strictly required for activation. This finding is consistent with the documented observation that deletion of *retS* alone is sufficient for GacS activation *in vivo* (9, 10, 12, 27).

For further biochemical studies, a recombinant CitAGacS_649_ protein, equivalent to GacS_649_, was expressed and purified. The affinity of both proteins for RetS_HK_ was measured using MST. Both CitAGacS_649_ and GacS_649_ yielded virtually identical apparent dissociation constants of 10 μM, further demonstrating their functional equivalence (Fig. 4*B*). The measured affinity between the two enzymes is notably weaker than reported in two prior studies, where, depending on the constructs, the dissociation constants ranged from 20 to 800 nM (9, 27). In part, the difference might be explained by differences in reaction buffers and reaction temperatures, 37 °C in our experiment *versus* the 25 °C used in the prior studies. However, the major reason for the differences is likely the inclusion of additional amino-terminal GacS domains. The present study constitutes the first instance, where binding was measured in the presence of the entire GacS HAMP domain, the TM helices, and the PDC domain. RetS and GacS are known to interact through their DHp domains, this interaction necessarily has to force apart the GacS dimer at the DHp site to accommodate RetS. Our HDX-MS data show that this conformational change in the DHp domain also causes conformational changes in GacS_HAMP_, GacS_TM_, and periplasmic GacS_PDC_. We therefore speculate, the larger measured dissociation constant for the RetS_HK_-GacS_649_ interactions reflects the additional energy required to cause these conformational changes in our more complete GacS construct; thus, explaining why prior experiments with GacS constructs lacking the amino-terminal domains appear to show tighter RetS binding.

To determine if ligand binding to the PDC domain also directly targets RetS-GacS interactions, the affinity between CitAGacS_649_ and RetS_HK_ was also measured in the presence of 10 mM sodium citrate. Citrate caused an almost five-fold decrease in the affinity of CitAGacS_649_ for RetS_HK_, thus providing the first direct evidence that the GacS ligand negatively impacts RetS-GacS affinity (Fig. 4*B*).

We experimentally examined underlying conformational changes associated with citrate binding to CitAGacS_649_ using HDX-MS (Fig. 4*C*). A comparative analysis of CitAGacS_649_ performed in the absence and presence of 10 mM citrate showed a dramatic impact on H/D exchange throughout the entire PDC domain consistent with the expected contraction of the binding pocket (46). Changes are also apparent on the periplasmic side of the lipid–water interface of the N-terminal TM helix. For example, residues 15 to 31 of TM-1 and 175 to 186 of TM-2 show decreased solvent accessibility in the citrate-complex. Yet, peptides linked to the cytosolic water–lipid interface show no change in deuterium uptake. Such a change might have been expected if citrate were to elicit a piston-type motion (39, 40, 51). Instead, structural changes in TM-1 and TM-2 are more consistent with a horizontal compaction of the membrane-embedded four-helix bundle. Strikingly, H/D exchange levels in the HAMP, S-helix, and HK regions were unaffected by citrate binding (Fig. 4*C*). This observation is consistent with *in vivo* data showing deletion of the retS gene is sufficient for GacS activation independent of bacterial growth stage (10). Notably, our autophosphorylation data also show significant activity of CitAGacS in the absence of citrate (Fig. 4*A*). Both results suggest that GacS is held inactive by RetS but can access the active conformation when RetS is not present irrespective of the presence of the ligand.

### RetS binding increases the affinity of CitAGacS_649_ for citrate

**Figure 5:**
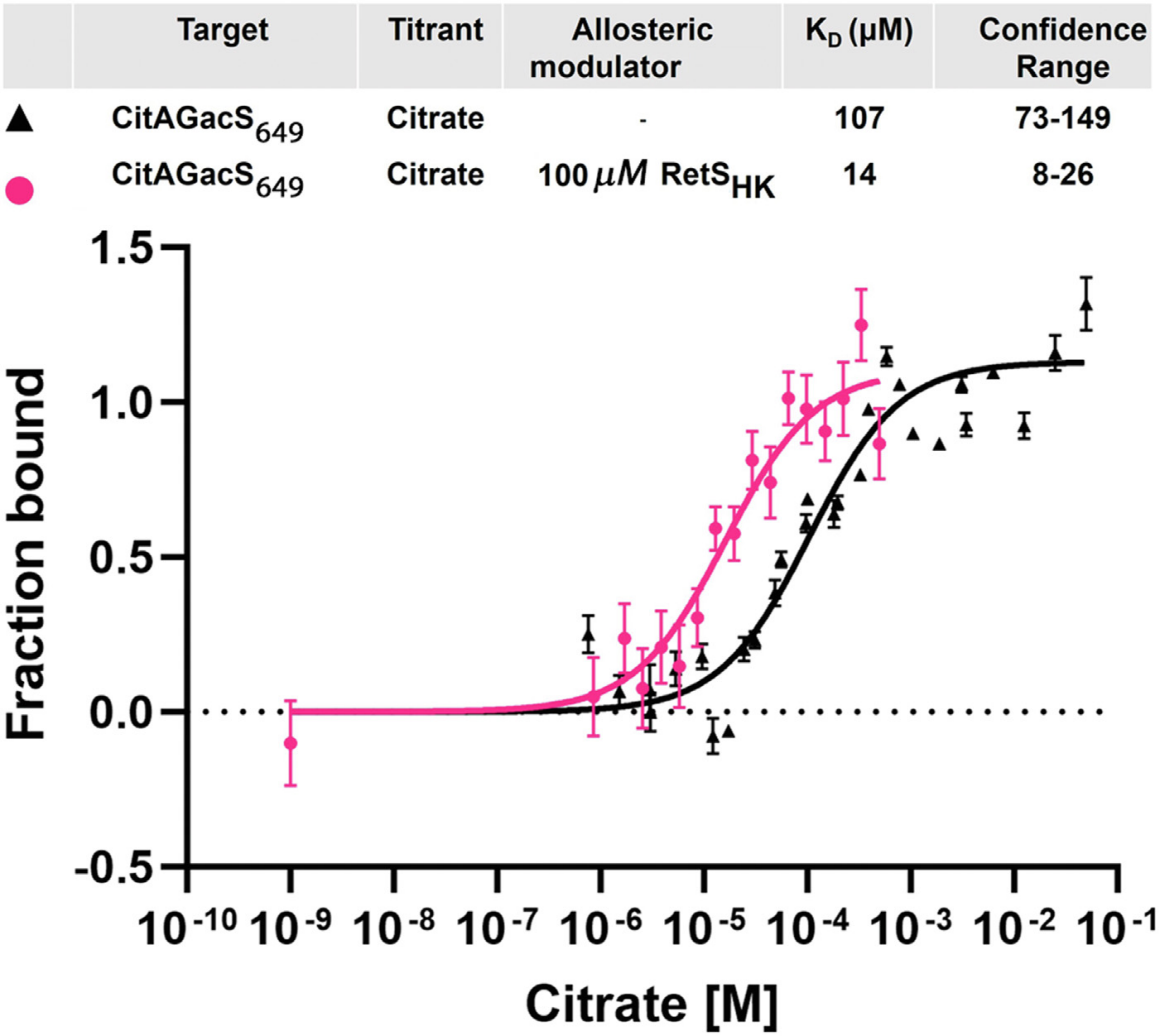
**MST analysis of CitAGacS_649_ binding to citrate, in absence and presence of RetS_HK_**. Each data point is representing the mean values average of triplicate experiments, with error bars representing the standard deviation (SD).

Because the natural GacS ligand is unknown, the CitAGacS_649_ protein was used to further investigate the hypothesis that RetS allosterically modulates the affinity of GacS for its extracellular ligand. Citrate binding to CitAGacS_649_ was measured in the absence and presence of excess RetS_HK_ using MST. In the absence of RetS_HK_, CitAGacS_649_ bound citrate with a dissociation constant of 107 μM (Fig. 5). When the MST measurement was repeated in the presence of 100 μM RetS_HK_, the dissociation constant for binding of CitAGacS_649_ to citrate was reduced to 14 μM, suggesting RetS is a positive allosteric modulator of GacS ligand binding (Fig. 5). To our knowledge, this constitutes the first experimental evidence that inside-out signaling may occur in bacterial signaling histidine kinases. In the context of the RetS/GacS system, this finding suggests that the binding of the elusive ligand may require priming of the GacS sensor through RetS, an observation that might prove critical to finally discovering its identity. The dissociation constant of primed CitAGacS_649_ is similar to the K_D_ = 5 μM reported for the isolated CitA_PDC_ domain (54). The binding affinity of full-length CitA for citrate has not been reported. The significance of structural context, however, is exemplified by a study comparing the properties of the isolated PDC domain of *Geobacillus thermodenitrificans* CitA with those of a construct that also includes the TM regions. Here, the addition of the TM helices caused an almost sixty-fold slowing of the binding kinetics (55).

## Discussion

The Gac/Rsm system has long been the model system for studying MKNs. Still many open questions remain, in particular why RetS uses three distinct mechanisms to regulate different stages of the GacS phosphorelay. In the first part of this study, we used HDX-MS analysis to examine the impact of RetS on GacS structure. Two prior studies investigated the original hypothesis that RetS binding prevents GacS trans-autophosphorylation by fully separating the GacS dimer (25, 27). Both groups used truncated constructs of GacS lacking the TM region, GacS_PDC_, as well as part of GacS_HAMP_ in their biochemical assays. Interestingly, while FRET-based binding data indicated the GacS dimer remains intact when RetS_HK_ binds, Fadel *et al*., using the exact same constructs, observed a full dissociation of the GacS dimer in their measurements (27). Because constructs used in prior studies lacked the full HAMP domain, the TM helices, and the PDC regions, we closely examined these regions for evidence of a dissociation of the predicted dimerization interface upon RetS_HK_ binding. The data are consistent with a model wherein GacS_HAMP_ and the extracellular GacS_PDC_ domain retain their dimerization interfaces in the presence of RetS. However, further verification is needed because of the limited timescale of the experiment and uncertainty associated with predicting the dimerization interface in the periplasm. Because the natural ligand of GacS_PDC_ is still unknown, we used a CitAGacS chimeric protein to study the impact of ligand binding on RetS-GacS interactions and autokinase activity. The observed impact of ligand binding is distinct from the role of the ligand in canonical systems, where ligand binding only stimulates SK autophosphorylation. CitAGacS was found to have substantial autokinase activity in the absence of the ligand, but citrate did have a positive effect that was statistically significant at later timepoints. Conformational changes induced by citrate binding appear to stabilize the active conformation of the histidine kinase region. In this context, a study of Dr DeGrado’s group is interesting, because it shows that conformational coupling between different domains of the related *Escherichia coli* signaling kinase PhoQ may be variable. For example, there are two distinct equilibria for Mg^2+^ ion binding (52, 53). Similarly, we observe weak conformational coupling between the citrate binding domain of CitAGacS and the HK region in absence of RetS. Collectively, a model emerges wherein citrate promotes CitAGacS activation by stabilizing the active conformation of the enzyme. However, as the enzymatic assay also demonstrates, CitAGacS can access the active conformation even without the ligand, albeit with slightly reduced frequency as long as RetS is also absent.

Analysis of HDX-MS data revealed an impact of citrate on solvent accessibility in PDC domain and the TM regions, but no significant impact on the CitAGacS histidine kinase region. This observation is consistent with the observed significant autokinase activity of CitAGacS in the absence of citrate and suggests that the ligand primarily targets RetS-GacS interactions by stabilizing the GacS homodimer. Such a model also explains why deletion of *retS* is sufficient for GacS activation *in vivo* as reflected in the hyper-adhesive phenotype of *retS*^*-*^ *P*. *aeruginosa* strains (8, 10, 12).

Our initial HDX-MS measurements indicate that RetS alters the conformation of the GacS_HAMP_ and sensory domains. This observation is significant because, in different contexts, HAMP domains have been shown to mediate inside-out cross-membrane signaling. Most relevant to the present study is the example of methyl-accepting chemotaxis proteins (MCPs). Here, HAMP domains facilitate bidirectional signaling to not only direct a chemotactic response to an extracellular ligand but also mediate adaptation, a process whereby the attractant affinity of the MCP is modulated through intracellular methylation and demethylation (32, 35). The cyclic-di-GMP sensor LapD from *Pseudomonas fluorescens* also utilizes inside-out signaling to regulate proteolytic activity in the periplasm (56). In SKs, discrete HAMP domain conformations have been associated with kinase ON and OFF states, although more recent work suggests an alternative model wherein ON/OFF states are linked to rigid *versus* relaxed conformations (33, 39, 40, 41). SK and MCP HAMP domains map to distinct phylogenetic branches. SK HAMP domains show strong evolutionary coupling with the kinase region, whereas MCP HAMP domains appear to be highly interchangeable (42). Because they are phylogenetically distinct and inside-out signaling had never been reported for SKs, we speculated that SK HAMP domains may have lost the capacity for bidirectional signaling (42, 43). Yet, the impact of RetS_HK_ on conformations of GacS_HAMP,_ the transmembrane regions, and the PDC domains raises the intriguing possibility that such signaling may indeed occur within the RetS/GacS-linked MKN. The binding studies with the chimeric CitAGacS construct provide further experimental support for an allosteric coupling between RetS binding and extracellular signal perception. It remains to be seen whether or not inside-out signaling is a unique feature of GacS, of SKs in MKNs, or universally conserved across the signaling histidine kinase family. The successful creation of a bidirectionally signaling chimeric protein suggests that, at least the ligand binding affinity of PDC-type sensory domains is intrinsically tunable through conformational coupling. Precisely how receptor affinity is modulated remains to be determined. The TM-1 helix is part of a much larger helix extending into the periplasm to form the backbone of GacS_PDC_. This helix is packed tightly against the backside of the contracting beta-sheet (Fig. S5), and, unsurprisingly, the equivalent region was significantly impacted by citrate binding in CitAGacS_649_. TM-2 connects directly to the C-terminal end of the final beta strand (Fig. S5). Therefore, affinity tuning may be facilitated by a combination of altering the position of TM-1 and by pulling on strand β-5 through TM-2.

**Figure 6:**
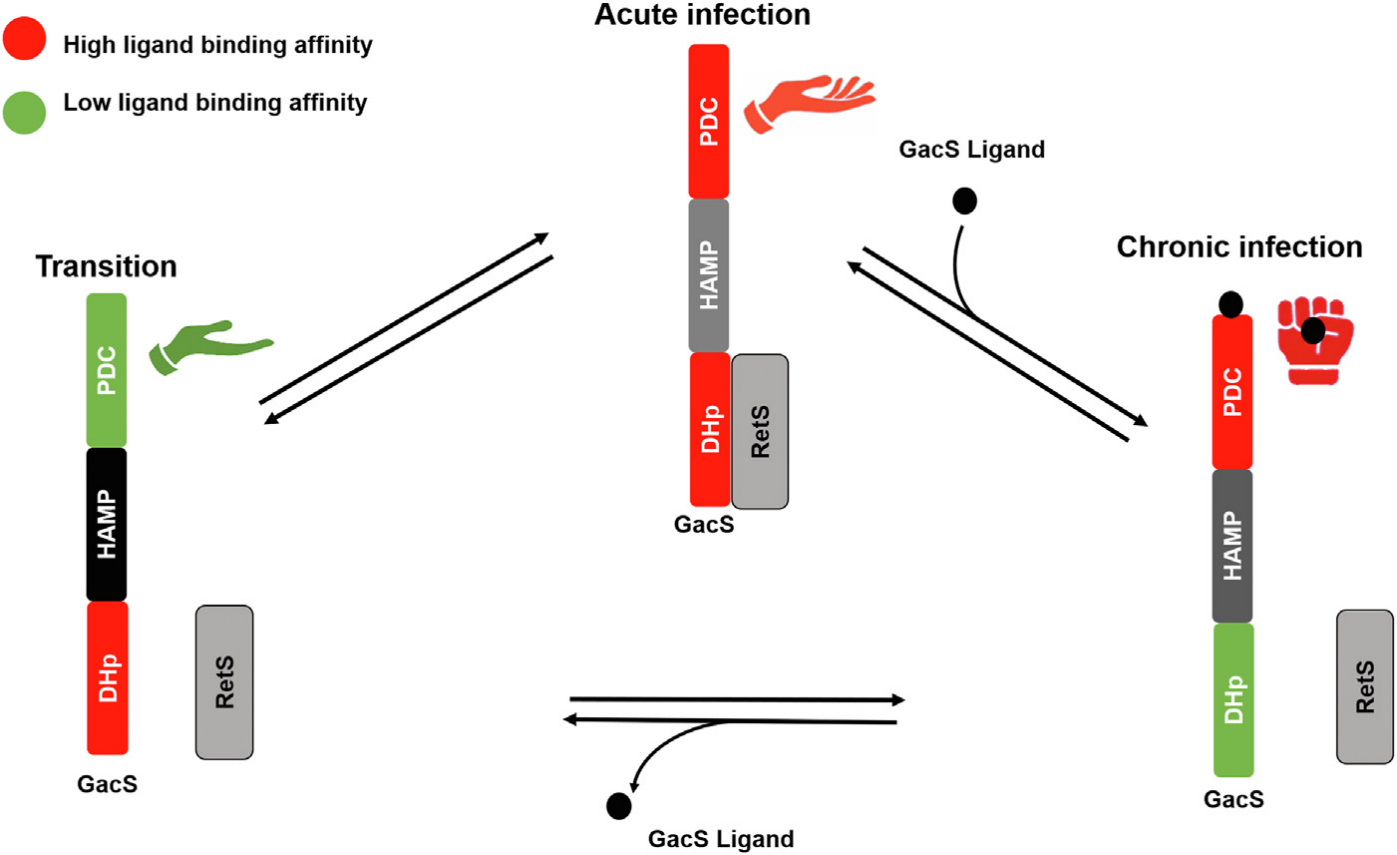
**A proposed dynamic cycle illustrates three assignable conformations of GacS, highlighting three significant domains**. The DHp and PD domain of GacS bind to RetS_DHp_ and putative ligand(s), respectively. It is hypothesized that GacS_HAMP_ plays a crucial role in transducing signals resulting from ligand or RetS_HK_ binding. The specific sequence of conformational changes involved in this cycle remains unknown.

The striking observation that RetS_HK_ allosterically promotes citrate binding to CitAGacS whereas citrate weakens RetS_HK_-CitAGacS interactions is difficult to explain with a simple four-state model. The work of Dr DeGrado’s group on the Mg^2+^-ion sensor PhoQ also suggests that the conformational landscape of multidomain histidine kinases is vastly more complex (52, 53). In this study, the authors uncovered complex allosteric coupling between the individual domains involving ten different conformational states. Therefore, rather than envisioning three or four conformations, multidomain signaling histidine kinases such as GacS likely have access to a complex conformational landscape to facilitate fine-tuned signaling. This is perhaps the reason why RetS uses three distinct mechanisms to interfere with GacS signaling at different stages of the phosphotransfer chain (23). We were able to identify only a single precedent for such unusual bi-directional allosteric coupling: Binding of vitamin B_12_ transporter BtuB to its intracellular partner protein TonB is allosterically promoted by vitamin B_12_. In turn, TonB binding triggers a conformational change in BtuB that reduces the affinity for vitamin B_12_ to cause its release into the cytosol (57). Although the conformational landscape of GacS is expected to be more complex, the present study revealed three distinct major conformations for GacS (Fig. 6): Apo-GacS with low affinity for the ligand but high affinity for RetS. RetS-bound GacS with high affinity for the ligand, and a ligand-bound GacS wherein the concomitant contraction of the PDC domain stabilizes the CitAGacS homodimer to reduce the affinity for RetS. The latter two conformations may be the more prevalent in the bacterial cell, with the RteS/GacS complex promoting planktonic growth, while the active, ligand-bound GacS orchestrates the transition to biofilm formation. Based on the observed binding behavior, ligand-bound GacS appears to be locked in the active conformation, perhaps to commit the cell toward biofilm formation. The identity of the GacS ligand remains unknown. The present study suggests that a successful *in vitro* screening strategy may require the inclusion of RetS.

In summary, this study establishes an important precedent for the inside-out signaling mechanism among SKs. More examples are likely to emerge at least among SKs in MKNs. RetS, for example, has its own sensory domain that interacts with at least two distinct carbohydrate-based ligands with opposite effects (29, 30). The compact domain architecture of RetS suggests that it is quite possible that GacS binding is not only modulated by these ligands but also impacts extracellular signal sensing of RetS. GacS and other hybrid SKs also feature pseudo response regulator domains, which regulate kinase activity presumably through binding of a small molecule ligand (27, 58). Whether or not these interactions impact extracellular signal perception has not been explored.

## Experimental procedures

### Recombinant protein expression plasmid design and construction

The plasmid construct for the expression of RetS_HK_ (residues 413–649) from the pDEST-HisMBP plasmid was created previously (12). All cloning primers are listed in Table S1. Table S2 lists the recombinant DNA used in this study. Expression plasmids for the production of GacS_649_, CitAGacS_649_, and CitAGacS were constructed in the present study. When expressed, all three proteins contain non-cleavable c-terminal octa-histidine tags. In the chimeric proteins, codons 42 to 158 for GacS_PDC_ have been replaced by codons 55 to 173 of CitA_PDC_. Genes of the truncated proteins contain codons for the N-terminal regions up to residue 649 of the native *gacS* sequence. For the expression of the GacS_649_ protein, the section of the *gacS* gene encoding residues 1 to 649 was amplified *via* PCR from a previously created *pDEST-HisMBP-gacS* vector containing the entire gacS gene using KpnIgacS1_F and His8-STOPBamH1GacS649_R primers. The PCR product was inserted into the arabinose-inducible pWarf(+) plasmid *via* restriction cloning to create *pWarf-gacS*_*649*_His_8_. The NEB Gibson Assembly Cloning Kit (New England Biolabs) was used to construct the *pHERD20T-citAgacS* plasmid used for the *in trans* expression of the corresponding protein in *P*. *aeruginosa*. Previously generated synthetic *pHERD20T-gacS* and *pHERD20T-citAretS* vectors served as templates. The NEB Gibson Assembly Cloning Kit (New England Biolabs) was also used for making the expression plasmid pWarf-citAgacS for full-length *citAgacS*. pWarf-citAgacS was used as a template to generate *pWarf-citAgacS*_-Full length_His_8_ and *pWarf-citAgacS*_649_His_8_. Primers KpnIgacS1_F and His8-STOPBamH1GacS649_R were used to construct *pWarf-citAgacS*_649_His_8_. For the expression of full-length CitAGacSHis_8_, primers KpnIgacS1_F and His8-STOPBamH1GacS_R were used. All constructs were verified *via* DNA sequencing.

### Recombinant protein expression and purification

Expression of *pWarf-gacS*_649_His_8,_ *pWarf-citAgacS*_649_His_8,_ and *pWarf-citAgacS*His_8_ follow the same protocol. Expression plasmid containing Lemo21 (DE3) cells (New England Biolabs) were cultured in 6 L Lennox broth (LB) supplemented with 50 μg/ml kanamycin, 30 μg/ml chloramphenicol, and 1% dextrose. The culture was incubated at 37 °C with shaking at 250 rpm until reaching an OD_600_ of 0.5 to 0.7. At this stage, induction of protein expression was initiated by adding 1 mM isopropyl β-D-1 thiogalactopyranoside (IPTG) and incubating the cells under shaking at 18 °C for 18 h. Cell harvest was conducted by centrifugation at 6000 x g for 20 min at 4 °C. Cell pellets were resuspended in buffer F (The compositions of the buffers are provided in Table S3) supplemented with 1 mM Phenylmethylsulfonyl fluoride (PMSF) and 1 mg/ml lysozyme. Cells were lyzed *via* sonication and the pellet collected through ultracentrifugation at 190,000×*g*. The pellet was resuspended in buffer F plus 2.5% Elugent (Millipore Sigma) detergent. For CitAGacS, Elugent was replaced by 2% DDM (n-dodecyl β-D-maltoside) detergent. Subsequently, the suspension was incubated at 4 °C for 2 h with gentle agitation. The residual pellet was removed through centrifugation for 1 h at 190,000 x g and 4 °C. Purification of all three proteins followed the exact same steps. Ni-NTA affinity chromatography was employed using two 5 ml Ni-NTA Superflow columns (Qiagen) and a 20 ml linear gradient elution with Ni-NTA buffer H containing 0.05% DDM detergent. Purification was monitored *via* SDS-PAGE analysis. Peak fractions were further purified on a 26/60 Superdex 200 column (GE Life Sciences) pre-equilibrated with GacS_649_ gel filtration buffer (buffer I). SDS-PAGE was again employed to corroborate protein purity. Gel filtration column elution profiles and SDS-PAGE images of peak fractions of purified CitAGacS, CitAGacS_649_, and GacS_649_ are provided in supplementary figure (Fig. S6). Expression and purification of RetS_HK_ followed previously published protocols (25).

### Crystal violet biofilm assay

We evaluated exopolysaccharide (EPS) production in *P*. *aeruginosa* PAO1 strain using the crystal violet biofilm assay. The *gacS*^*-*^-PAO1 strain was created by Dr Jenal’s group in a prior study and kindly shared with us (59). Initially, strains were cultured on LB agar plates containing 300 μg/ml carbenicillin. Subsequently, individual colonies were used to inoculate LB broth containing 300 μg/ml carbenicillin, and the cultures were incubated overnight with agitation at 250 rpm at 37 °C. Strains were subcultured into modified M63 media containing 0.5% arabinose and 300 μg/ml carbenicillin, and again incubated overnight with agitation at 250 rpm at 37 °C. The OD_600_ of the cultures was adjusted to 0.05 using modified M63 media containing 0.5% arabinose, 300 μg/ml carbenicillin, and grown with or without 20 mM sodium citrate pH 7.4. Subsequently, 100 μl of each culture was dispensed into individual wells of 96-well plates (Corning # 2797). The plates were covered with aluminum foil and incubated at 37 °C for 8 h under gentle rocking. Media were then removed *via* pipette and wells were washed twice with water. Wells were then stained and incubated with 0.1% crystal violet for 10 min. The stained cells were subsequently washed with water *via* pipette. The crystal violet-stained cells were solubilized in 125 μl of 30% acetic acid for 15 min 100 μl of the solubilized solution was transferred to a 96-well plate (Corning # 3370) and the absorbance was measured at λ = 600 nm using a Tecan M200 plate reader. The experimental results shown in Figs 3*C* and S3 represent the average of biological and technical triplicates.

### Microscale thermophoresis

Microscale Thermophoresis (MST) measurements were carried out using a Monolith NT.115^PICO^ system (NanoTemper Inc.). GacS_649_ and CitAGacS_649_ proteins, solubilized in buffer J, were fluorescently labeled with amine-reactive AlexaFluor 488 (Life Technologies) following the manufacturer's instructions. The compositions of all buffers are provided in Table S3. For the binding studies, the proteins were then diluted to 80 nM and the detergent concentration was reduced to 0.007% DDM. In addition to optimizing the detergent concentration, improved signal-to-noise and more rapid equilibration were observed at 37 °C. Therefore, all MST measurements were carried out at that temperature. For the affinity measurements of GacS_649_ and CitAGacS_649_ to RetS_HK_, serial dilutions of RetS_HK_ from 140 μM to 1.06 nM were prepared. Measurements were carried out in the presence and absence of 10 mM sodium citrate. Data analysis was performed using the associated company software.

Subsequently, we examined CitAGacS_649_ binding affinity to different concentrations of sodium citrate ranging from 0.1 μM to 12 mM in the absence and presence of 100 μM RetS_HK_. All experiments were conducted using premium treated capillaries (NanoTemper) in the high IR-laser power setting. During the scans, laser on-time intervals were set at 15 s, and off-time intervals at the beginning and end were set to 5 s. Each experiment was repeated three times.

### *In vitro autophosphorylation assay using* dot blot autoradiography

The *in vitro* autophosphorylation assay closely adhered to the steps outlined in the published general protocol (60). Full-length CitAGacS-His_8_ was used for the assay. The final concentration of CitAGacS in the 100 μl reactions was 5 μM. Enzyme, nucleotide, and citrate stock solutions were all prepared in a modified buffer J containing only 0.015% DDM as opposed to 0.03% DDM. Prior to the addition of ATP, we ensured that all reactions contained the exact same amount of enzyme by measuring the absorption of each solution at 280 nm using a nanodrop spectrophotometer (Thermo Scientific, Inc.). The final ATP concentration in each reaction was 0.5 mM and 0.1 μCi/μl γ^32^P]-ATP (Revvity Health Sciences, Inc.). Reactions were incubated at 37 °C, and 10 μl aliquots collected and quenched at 0, 5, 10, 20, and 40 min timepoints. The aliquots were pipetted onto 0.2 μm nitrocellulose membrane. Subsequent steps followed the published protocol exactly, with the exception that, *in lieu* of a 96-well dot blot microfiltration apparatus, blots were washed five times in a Tupperware container for 10 min each time using 100 ml of 25 mM phosphoric acid. The membranes were then exposed to x-ray film, visualized by autoradiography, and quantified using ImageJ2 (61). Experiments were performed in triplicate.

### Hydrogen-deuterium exchange mass spectrometry (HDX-MS)

For HDX-MS, stock solutions of GacS_649_ (14 μM), CitAGacS_649_ (27 μM) and sodium citrate (100 mM) were prepared separately in GacS buffer I (12.5 mM Tris/TAPS pH 8.5, 125 mM NaCl,1 mM TCEP, 0.03% DDM). A stock solution of RetS_HK_ (350 μM) was prepared in RetS_HK_ buffer E (25 mM Tris pH 7.4, 125 mM NaCl, 1 mM TCEP, 2.5% glycerol). Prior to data collection, GacS_649_ or CitAGacS_649_ were diluted to a final concentration of 5 μM. An equal volume of RetS buffer (“Apo” sample), RetS_HK_ protein to final 34 μM RetS_HK_ concentration (“RetS Complex”) or sodium citrate prepared in GacS buffer I to a final concentration of 10 mM (“CitAGacS_649_-Citrate”) were added and reactions were incubated at room temperature for 30 min prior to storage at 0.1˚C for the HDX-MS experiment. We should note that protein solubility limited the concentration of the RetS_HK_ stock solution. Therefore, the concentration of RetS_HK_ in the final reaction was restricted to 34 μM. In the context of the measured dissociation constant, binding was therefore not fully saturated but more than 75% of GacS_649_ protein is in complex with RetS_HK_.

HDX-MS data collection and analysis were performed at the Biomolecular and Proteomics Mass Spectrometry Facility (BPMSF) of the University California San Diego using a Waters Synapt G2Si system with HDX-1 autosampler (Waters Corporation, Milford, MA). D_2_O buffer was prepared by lyophilizing GacS buffer and then re-dissolving it in an equivalent volume of 99.96% D_2_O (Cambridge Isotope Laboratories, Inc) just prior to use. Deuterium exchange measurements were performed in triplicate for all time points (0 min, 0.5 min, 1 min, 2 min, 5 min) as described previously (62). A solution of 3 M guanidine hydrochloride (50 μl, final pH 2.66) was added to each sample (50 μl) and incubated for 1 min at 1 °C to quench deuterium exchange and denature the protein. The 90 μl of quenched sample were injected into a 100 uL sample loop for in-line digestion at 15 °C *via* a pepsin column (Immobilized Pepsin, Pierce). Peptides were then captured on a BEH C18 Vanguard precolumn and then separated by analytical chromatography (Acquity UPLC BEH C18, 1.7 μm 1.0 × 50 mm, Waters Corporation) over 7.5 min using a 7 to 85% acetonitrile gradient containing 0.1% formic acid. Samples were then electrosprayed into the Synapt G2Si mass spectrometer. Data were collected in the Mobility, ESI + mode (mass acquisition range = 200–2000 (m/z); scan time = 0.4 s). An infusion of leu-enkephalin (m/z = 556.277) every 30 s was used for continuous lock mass correction (mass accuracy = 1 ppm for calibration standard).

To identify peptides, data was collected in mobility-enhanced data-independent acquisition (MS^E^), mobility ESI + mode. Peptide masses were determined from triplicate analyses, and resulting data were analyzed using ProteinLynx global server (PLGS) version 3.0 (Waters Corporation). We identified peptide masses using a minimum number of 250 ion counts for low energy peptides and 50 ion counts for their fragment ions, with the requirement that peptides be larger than 1500 Da. After identifying peptides in PLGS, peptides were analyzed with DynamX 3.0 software (Waters Corporation); relative deuterium uptake was calculated by comparison of the centroids of the mass envelope of the deuterated peptides with non-deuterated controls per previously reported methods (62), and used to obtain data for coverage maps. Data are represented as mean values ± SD of the three technical replicates due to processing software limitations, but we note that the LEAP robot provides highly reproducible data for biological replicates. Back-exchange was corrected for in the deuterium uptake values using a global back exchange correction factor (typically ∼25%) determined from the average percent exchange measured in disordered termini of varied proteins (63). Significance among differences in HDX data points was assessed using ANOVA analyses and T-tests with DECA (64). We generated deuterium uptake plots in DECA (github.com/komiveslab/DECA), with data plotted as deuterium uptake (corrected) *versus* time. An HDX-MS data summary table is shown in Table S4, and Source Data files are provided with the supplementary materials as Tables S5 and S6. Butterfly plots and coverage maps for the HDX-MS analyses are provided in supplementary figures Figures S7 and S8.

## Data availability

All data are included in the submission. HDX update analysis data are included in the article and/or supporting information.

## Supporting information

This article contains supporting information.

## Conflict of interest

The authors declare that they have no conflicts of interest with the contents of this article.

## Supporting information

Supplementary Fgures
