# Supplementary material for: Evidence of bidirectional transmembrane signaling by the sensor histidine kinase GacS from Pseudomonas aeruginosa
Source: J Biol Chem. 2025 Apr 23;301(6):108521. doi: 10.1016/j.jbc.2025.108521 (PMC12148439; doi:10.1016/j.jbc.2025.108521)
Supplement: Supplementary Fgures [file mmc1.docx]

**Table S1- cloning primers**

| F1_fwd-pHERD20T-chimeric | taagaattcgagctcggtaccatgttcaaggatctcggcatcaagg |
| --- | --- |
| F1_rev-pHERD20T-chimeric | gcccgacctgggcgcgcatgtcggacag |
| F2_fwd-pHERD20T-chimeric | catgcgcgcccaggtcgggcaacgggcg |
| F2_rev-pHERD20T-chimeric | cgcgcagcagcagttgctcaatggtatagcctaccgacac |
| F3_fwd-pHERD20T-chimeric | tgagcaactgctgctgcgcggatatcgc |
| F3_rev-pHERD20T-chimeric | taaaacgacggccagtgccaagctcagagttcgctggagtcg |
| KpnIgacS1_F | gtggtggtggtggtgatgatgatg gcg |
| His8-STOPBamH1GacS649_R | gtggtggtggtggtgatgatgatg gcg |
| F1_fwd-pWarf-chimeric-full length | atgttaagaattccgggtacatgttcaaggatctcggcatcaagg |
| F1_rev-pWarf-chimeric-full length | gcccgacctgggcgcgcatgtcggacag |
| F2_fwd-pWarf-chimeric-full length | catgcgcgcccaggtcgggcaacgggcg |
| F2_rev-pWarf-chimeric-full length | cgcgcagcagcagttgctcaatggtatagcctaccgacac |
| F3_fwd-pWarf-chimeric-full length | tgagcaactgctgctgcgcggatatcgc |
| F3_rev-pWarf-chimeric-full length | cctgaaacaagacttccaagtcagagttcgctggagtcg |

|  |
| --- |
|  |
| **Table S2- Recombinant DNA**   \| **Recombinant DNA** \| **Source** \| \| --- \| --- \| \| pDEST-HisMBP-RetS_HK_ \| Mancl et al., 2019 \| \| pDEST-HisMBP \| Invitrogen \| \| pHERD20T \| Qiu et al., 2018 \| \| pHERD20T-retS \| Mancl et al., 2019 \| \| pHERD20T-gacS \| Mancl, 2018 \| \| pHERD20T-citA-retS2 \| Kaler, 2021 via Genewiz \| \| pWarf-gacS_649_His_8_ \| This study \| \| pWarf-citAgacS_649_His_8_ \| This study \| \| pWarf-citAgacS-Full length -His_8_ \| This study \| \| pWarf-citAgacS-Full length \| This study \| \| pHERD20T-citAgacS \| This study \| |
|  |
|  |

**Table S3 - List of Buffers**

| **Buffer** | **Composition** | **Purpose** |
| --- | --- | --- |
| A | 50 mM tris pH 7.4, 500 mM NaCl, 5 % glycerol, 25 mM imidazole pH 7.4, 1 mM DTT | Cell resuspension, separation by binding to NiNTA column (used for RetS_HK_) |
| B | 50 mM tris pH 7.4, 500 mM NaCl, 5 % glycerol, 250 mM imidazole pH 7.4, 1 mM DTT | To elute from the NiNTA column (used for RetS_HK_) |
| C | 50 mM tris pH 7.4, 50 mM NaCl, 10 % glycerol, 1 mM DTT | Binding to IEC column (used for RetS_HK_) |
| D | 50 mM tris pH 7.4, 500 mM NaCl, 10 % glycerol, 1 mM DTT | Elution from IEC column (used for RetS_HK_) |
| E | 25 mM tris pH 7.4, 125 mM NaCl, 2.5 % glycerol, 1 mM TCEP pH 8.0 | Gel filtration column elution,  HDX-MS analysis (used for RetS_HK_) |
| F | 25 mM Tris/TAPS pH 8.2, 500 mM NaCl, 5 % glycerol, 25 mM imidazole pH 8.2, 1 mM DTT, 25mM MgCl2 | Cell resuspension (used for CitA GacS_649_, CitAGacS Full length, and  GacS_649_) |
| G | 25 mM Tris/TAPS pH 8.2, 500 mM NaCl, 5 % glycerol, 25 mM imidazole pH 8.4, 1 mM DTT, 5mM MgCl2, 0.05% DDM | Cell resuspension, binding to NiNTA column (used for CitA GacS_649_, CitAGacS Full length, and  GacS_649_) |
| H | 25 mM Tris/TAPS pH 8.2, 500 mM NaCl, 5 % glycerol, 350 mM imidazole pH 8.4, 1 mM DTT, 25mM MgCl_2_, 0.05% DDM | To elute from the NiNTA column (used for CitA GacS_649_, CitAGacS Full length, and  GacS_649_) |
| I | 12.5 mM Tris/TAPS pH 8.2, 125 mM NaCl, 1 mM TCEP, 0.03% DDM | Gel filtration column elution, HDX-MS analysis (used for CitA GacS_649_, CitAGacS Full length, and  GacS_649_) |
| J | 25 mM HEPES pH 8.2, 125 mM NaCl, 1 mM TCEP, 5mM MgCl_2_, 0.03% DDM | Gel filtration column elution, fluorescent labeling for MST analysis, *In vitro autophosphorylation assay* (used for CitA GacS_649_, CitAGacS Full length, and  GacS_649_) |

| **Table S4. HDX-MS DATA** |  |  |
| --- | --- | --- |
| **GacS_649_** |  |  |
| **Data Set** | **GacS_649_** | **GacS_649_:RetS_HK_** |
| HDX reaction details | 12.5 mM Tris/TAPS, 125 mM NaCl,1mM TCEP, 0.03% DDM, pD= 8.85@4 °C | 12.5 mM Tris/TAPS, 125 mM NaCl,1mM TCEP, 0.03% DDM, pD= 8.85@4 °C |
| HDX time course (min) | 0,0.5, 1, 2, 5 | 0,0.5, 1, 2, 5 |
| HDX control samples | Disordered section of WT NT protein | Disordered section of WT NT protein |
| Back-exchange (mean / IQR) | 21%/3.5% | 21%/3.5% |
| # of Peptides | 98 | 98 |
| Sequence coverage | 91.2% | 91.2% |
| Average peptide length / Redundancy | 14.3/ 2.35 | 14.3/ 2.35 |
| Replicates (biological or technical) | 3 (technical) | 3 (technical) |
| Repeatability | 0.084 (average standard deviation) | 0.113 (average standard deviation) |
| Significant differences in HDX (delta HDX > X D) | 0.25D (95% CI) | 0.25D (95% CI) |
|  |  |  |
| **CitAGacS_649_** |  |  |
| **Data Set** | **CitAGacS_649_** | **CitAGacS_649_:Citrate** |
| HDX reaction details | 12.5 mM Tris/TAPS, 125 mM NaCl,1mM TCEP, 0.03% DDM, pD= 8.85@4 °C | 12.5 mM Tris/TAPS, 125 mM NaCl,1mM TCEP, 0.03% DDM, pD= 8.85@4 °C |
| HDX time course (min) | 0.5, 1, 2, 5 | 0.5, 1, 2, 5 |
| HDX control samples | Disordered section of WT NT protein | Disordered section of WT NT protein |
| Back-exchange (mean / IQR) | 21%/3.5% | 21%/3.5% |
| # of Peptides | 189 | 189 |
| Sequence coverage | 95.6% | 95.6% |
| Average peptide length / Redundancy | 14.4/ 4.31 | 14.4/ 4.31 |
| Replicates (biological or technical) | 3 (technical) | 3 (technical) |
| Repeatability | 0.060 (average standard deviation) | 0.067 (average standard deviation) |
| Significant differences in HDX (delta HDX > X D) | 0.25D (95% CI) | 0.25D (95% CI) |
