# Supplementary material for: Evidence of bidirectional transmembrane signaling by the sensor histidine kinase GacS from Pseudomonas aeruginosa
Source: J Biol Chem. 2025 Apr 23;301(6):108521. doi: 10.1016/j.jbc.2025.108521 (PMC12148439; doi:10.1016/j.jbc.2025.108521)
Supplement: Supplementary Tables [file mmc2.docx]

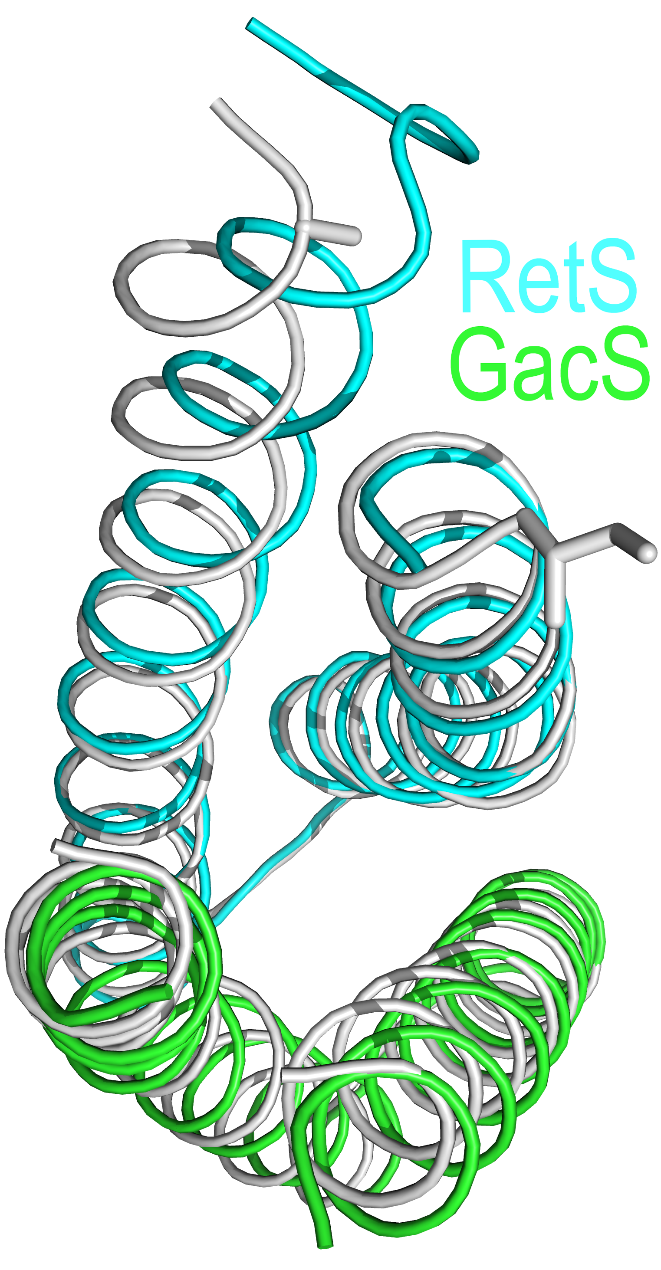


**Figure S1.** *Superposition of the homodimeric DHp-DHp interface from crystal structure of the GacS-HK protein (grey, PDB code: 7Z8N) and the heterodimeric interface from GacS_DHP_-RetS_HK_ complex (GacS_DHP_ green; RetS_HK_ turquoise; PDB code: 7N0E).*


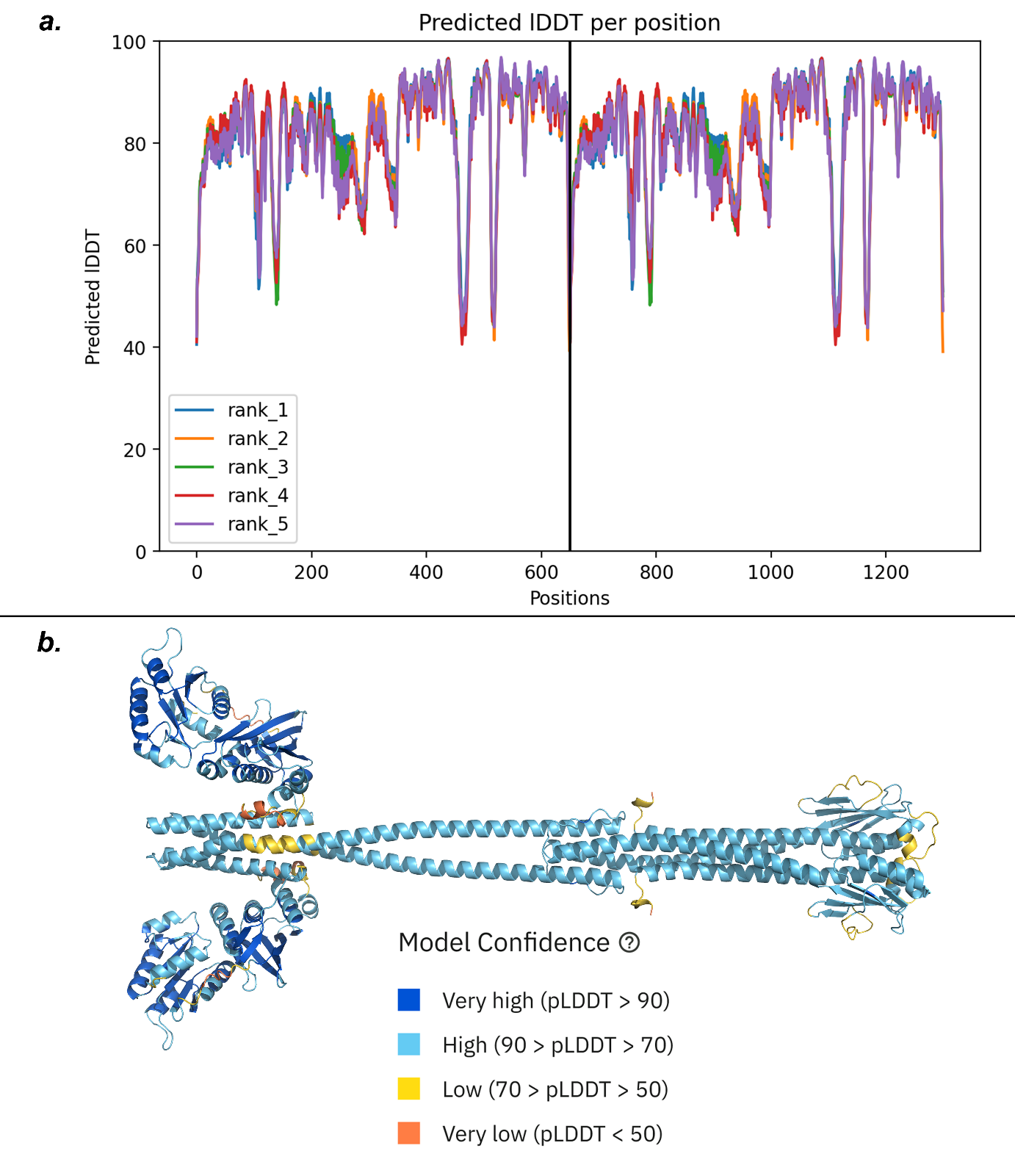


**Figure S2.** AlphaFold2-generated model of the GacS_649_ domain.


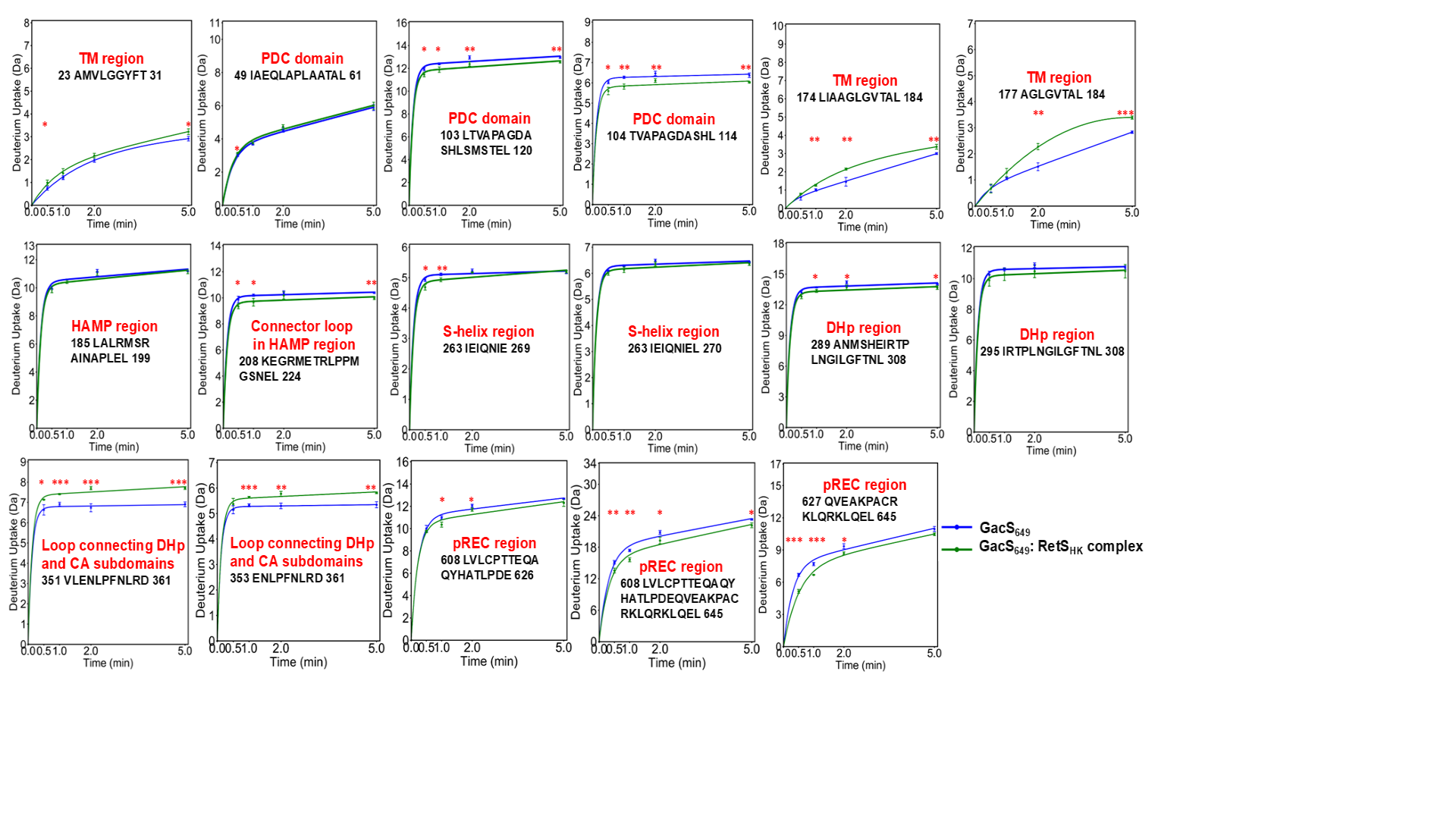


**Figure S3.** Additional uptake graphs of HDX analysis of the interactions between GacS_649_ and RetS_HK_ covering highlighted regions. **P<0.05, **P<0.01, ***P<0.001.*


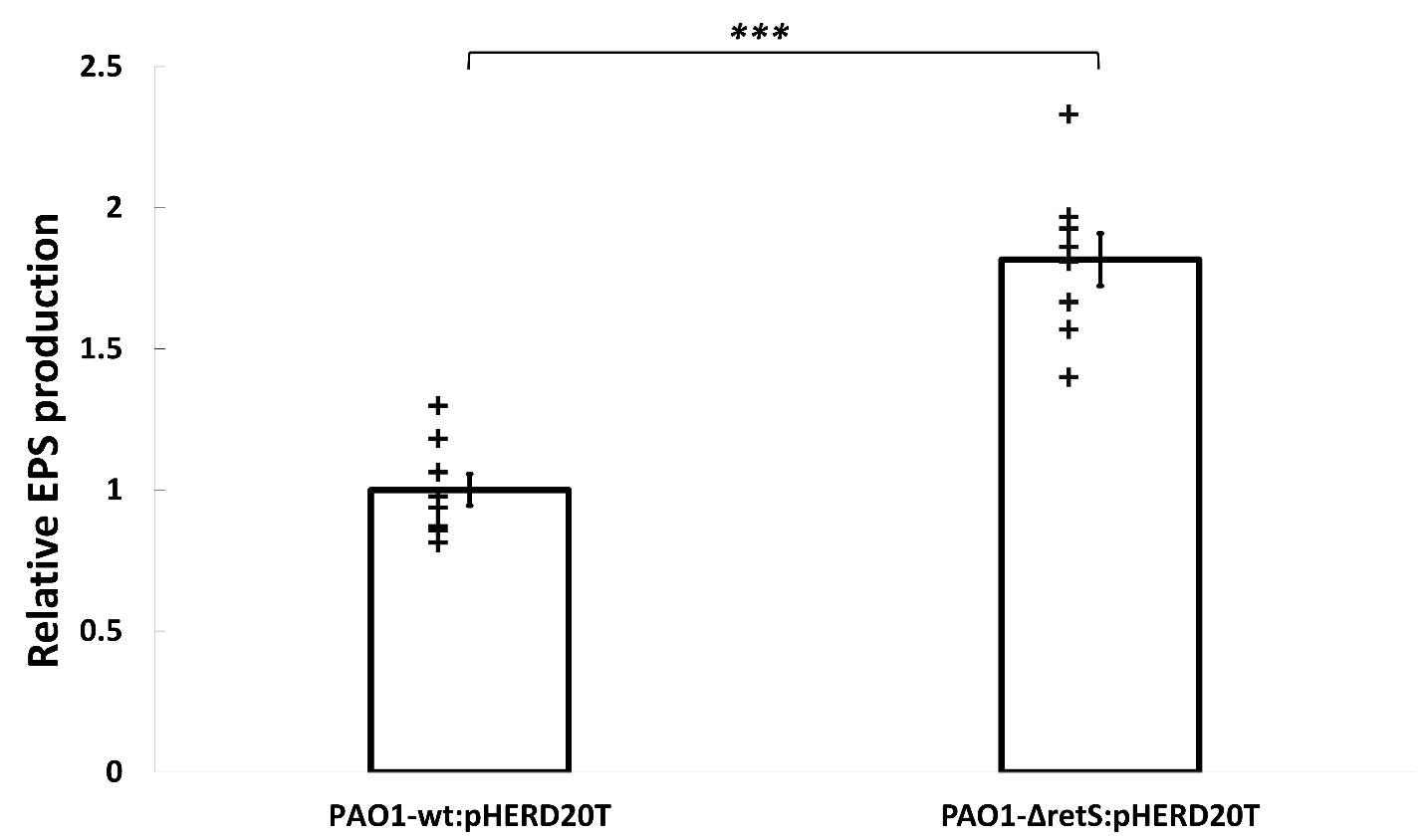


**Figure S4.** Results of an *in vivo* attachment assay monitoring exopolysaccharide production as proxy for biofilm formation comparing wt PAO1 and *retS ^-^*PaO1 strains. **P<0.05, **P<0.01, ***P<0.001.*


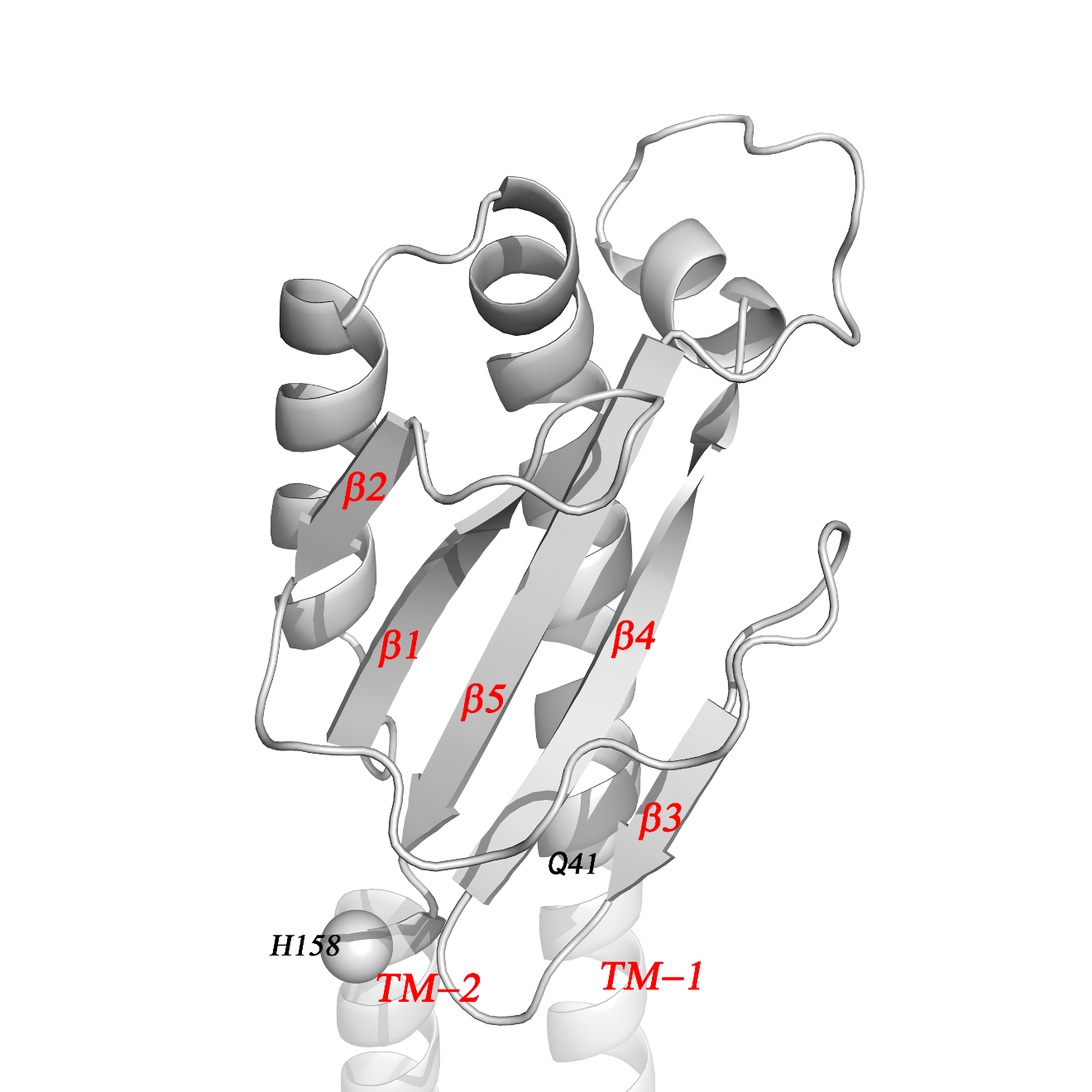


**Figure S5.** AlphaFold-generated model of the GacS_PDC_ domain


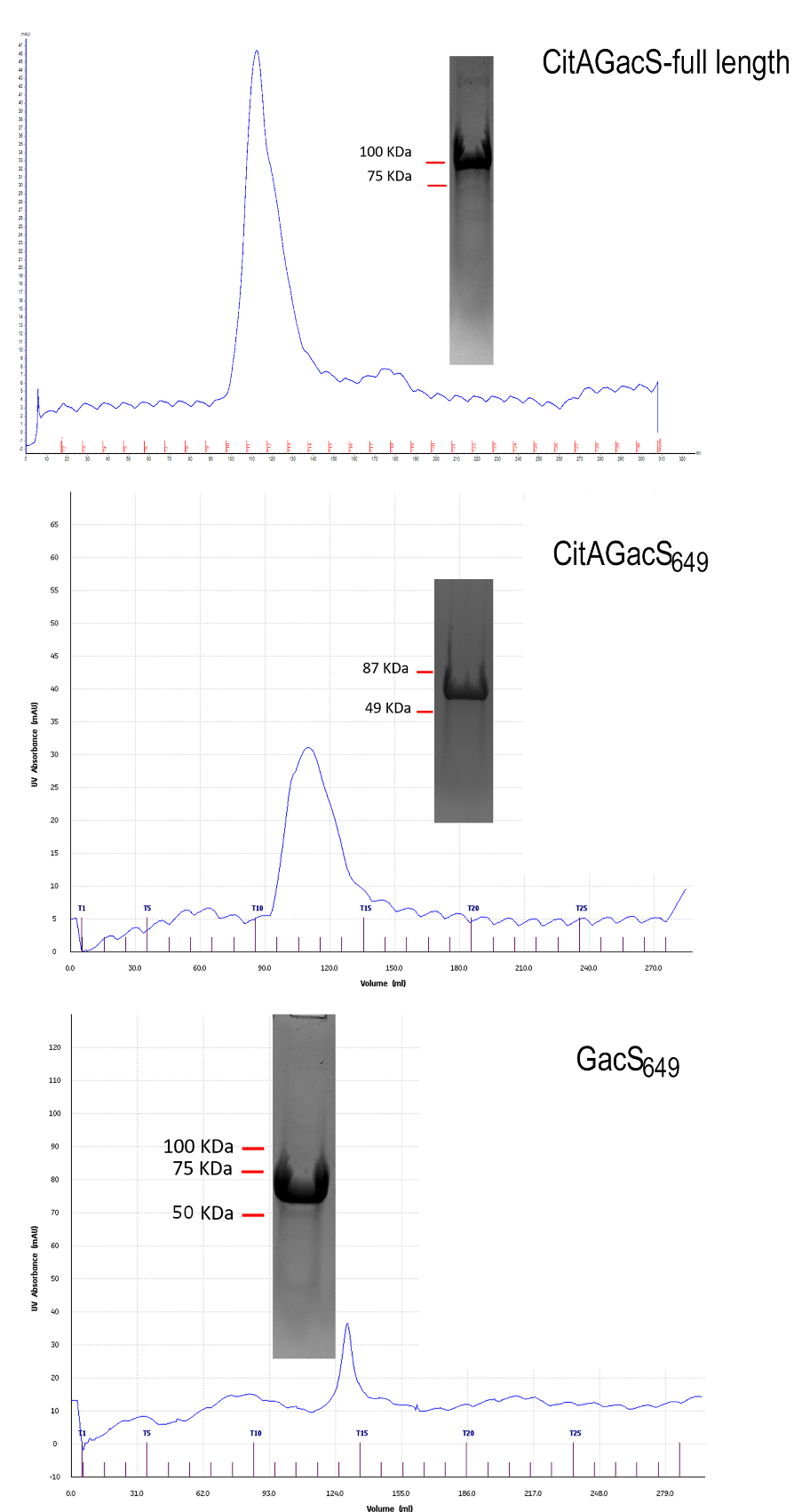


**Figure S6.** Gel filtration elution profiles of purified proteins and SDS-PAGE of peak fractions.


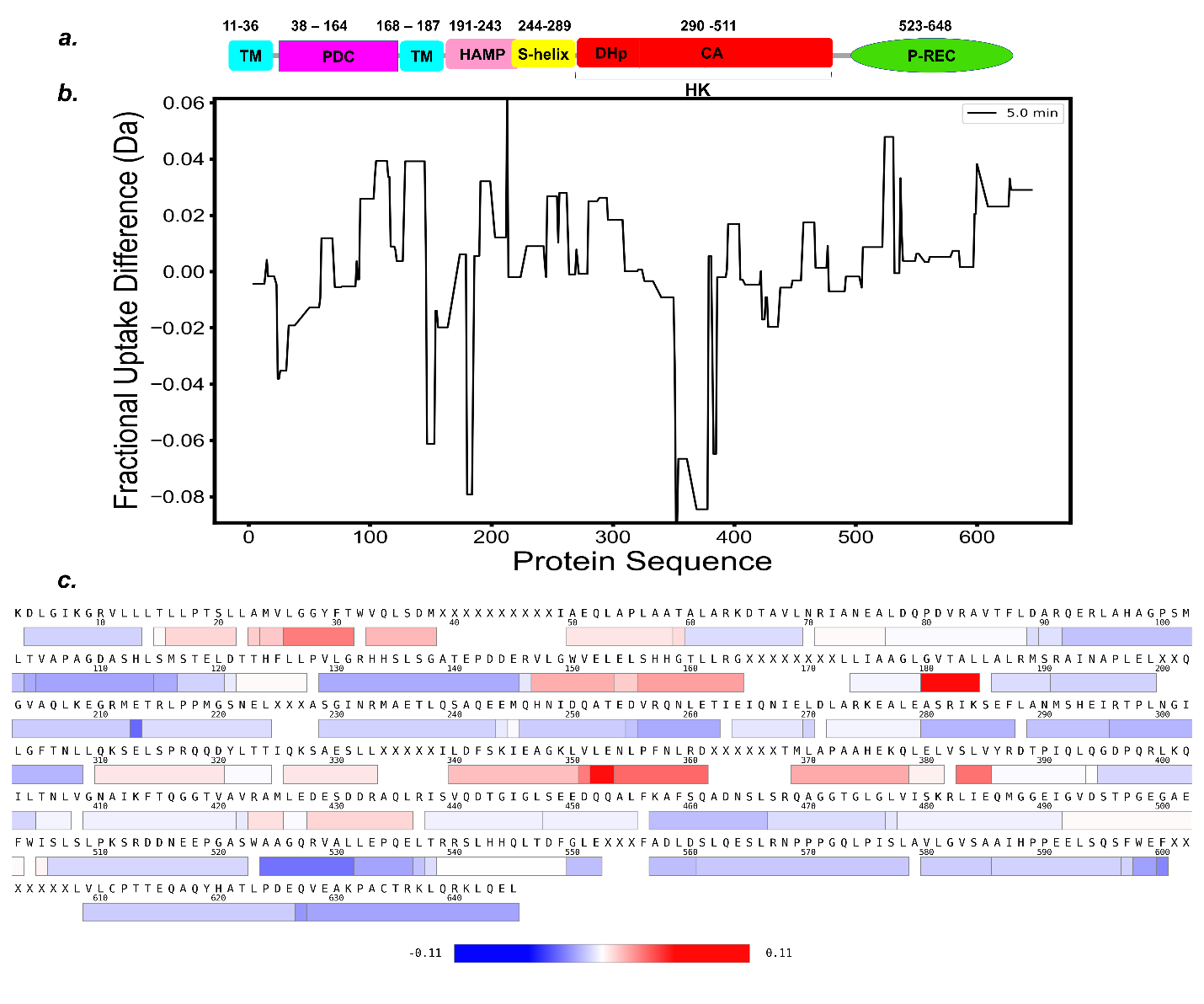
**Figure S7.** **a.** Schematic of GacS_649_ domain architecture with sequence numbers above. **b.** Plot of uptake differences of GacS_649_ in the presence of 30-fold molar excess of RetS_HK_. **c.** Coverage map of GacS_649_ color coded according to uptake differences of GacS_649_ in the presence of 30-fold molar excess of RetS_HK_. The statistical significance of the represented uptake differences depends on the variable standard deviations observed for the measurements of each peptide. For additional information please refer to tables S4 and S5.

_
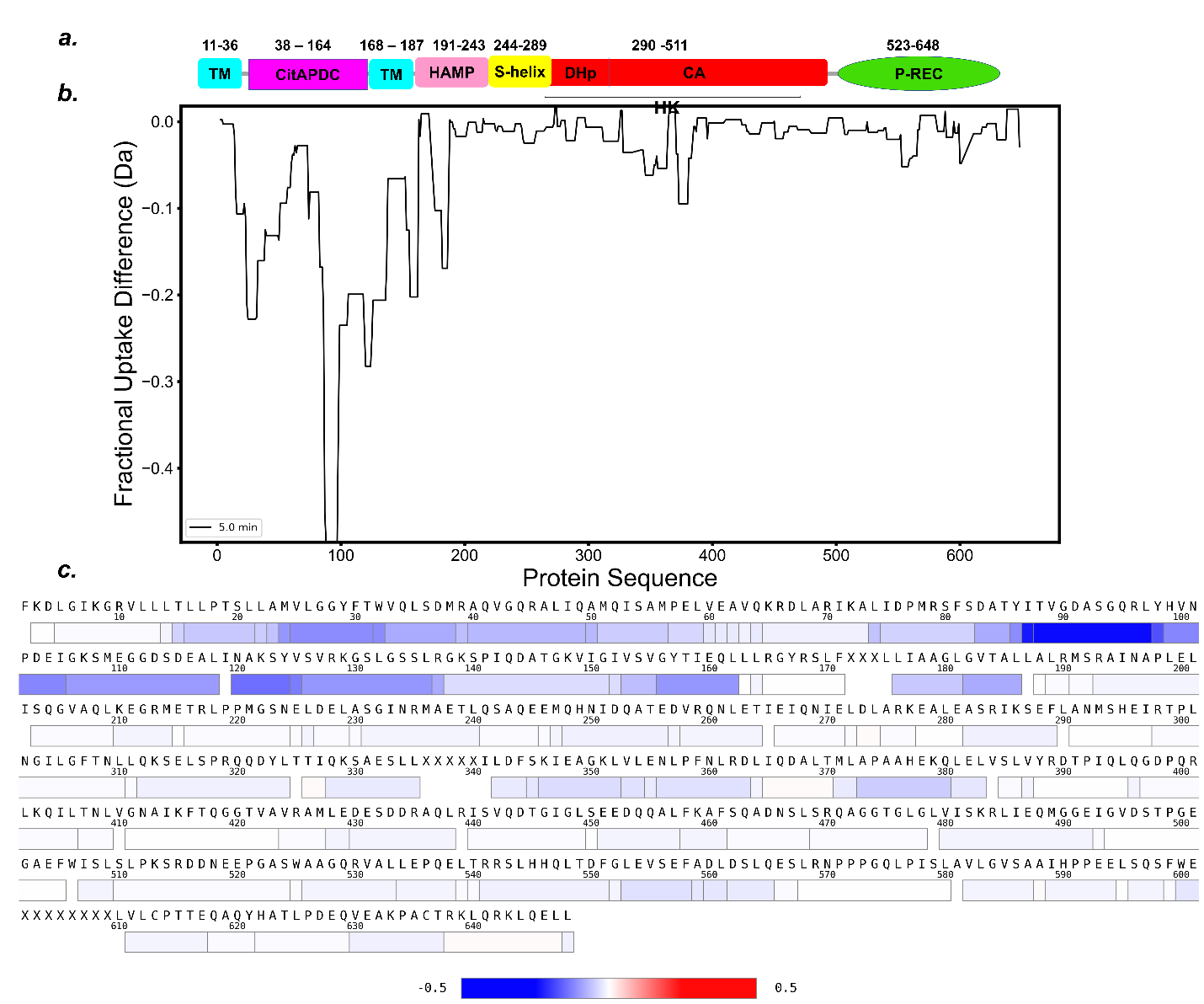
_

**Figure S8. a.** Schematic of CitAGacS_649_ domain architecture with sequence numbers above. **b.** Plot of uptake differences of CitAGacS_649_ in the presence citrate. **c.** Coverage map of CitAGacS_649_ color coded according to uptake differences of CitAGacS_649_ in the presence of citrate. The statistical significance of the represented uptake differences depends on the variable standard deviations observed for the measurements of each peptide. For additional information please refer to tables S4 and S6.
